# *In Planta* Localization of Endophytic *Cordyceps fumosorosea* in Carrizo Citrus

**DOI:** 10.3390/microorganisms9020219

**Published:** 2021-01-22

**Authors:** Ethan M. Doherty, Pasco B. Avery, Emily B. Duren, Liliana M. Cano, Lorenzo Rossi

**Affiliations:** 1 Horticultural Sciences Department, Indian River Research and Education Center, Institute of Food and Agricultural Sciences, University of Florida, Fort Pierce, FL 34945, USA; EDoherty@agcenter.lsu.edu; 2 Department of Entomology, Louisiana State University, Baton Rouge, LA 70803, USA; 3 Entomology and Nematology Department, Indian River Research and Education Center, Institute of Food and Agricultural Sciences, University of Florida, Fort Pierce, FL 34945, USA; pbavery@ufl.edu (P.B.A.); emilyduren94@gmail.com (E.B.D.); 4 Plant Pathology Department, Indian River Research and Education Center, Institute of Food and Agricultural Sciences, University of Florida, Fort Pierce, FL 34945, USA; lmcano@ufl.edu

**Keywords:** Carrizo citrange, *Cordyceps fumosorosea*, endophytism, entomopathogenic fungus, seed immersion, stem injection, sand column

## Abstract

Entomopathogenic fungi can be a useful resource for controlling insect vectors of citrus plant pathogens, such as the Asian citrus psyllid (*Diaphorina citri*) associated with huanglongbing or the citrus root weevil (*Diaprepes abbreviatus*) associated with the spread of *Phytophtora* spp. In this study, *Cordyceps fumosorosea* (*Cfr*) was investigated *in planta* as a potential endophytic entomopathogenic fungus and various inoculation techniques were used to determine if it would colonize the Carrizo citrange (*Citrus × insitorum*) seeds and plants. The four inoculation methodologies evaluated were seed soaking, stem injection, foliar spray, and soil drench. Seed immersion trials demonstrated that the roots of the Carrizo citrange plant can be inoculated successfully with *Cfr*. Stem injection, foliar spray, and soil drench also provided successful inoculation of *Cfr*. However, this fungus was only endophytic in the plant stem. Sand cores indicated that *Cfr* moved down through the sand column and was able to inoculate the roots. Given the prevalence of *Cfr* in the soil during the drench experiment, and that the fungus was able to colonize Carrizo citrange roots through seed immersion, this finding provides evidence of the potential endophytism of this fungus when applied to citrus plant species.

## 1. Introduction

Plant-associated microbes, including fungi, commonly occur in nature and may form endophytic relationships [1]. An endophytic fungus that lives within the plant may provide a benefit for the plant host, thereby forming a mutually beneficial relationship [2,3,4]. Endophytic entomopathogenic fungi (EEPF) may provide protection by being pathogenic to the arthropod pests that feed on the host plant [5]. For example, Quesada-Moraga et al. [6] found that damage to opium poppy (*Papaver somniferum* L.) by the poppy stem gall wasp, *Iraella luteipes* (Thompson) (Hymenoptera: Cynipidae) was reduced by the EEPF *Beauveria bassiana* (Bals.-Criv.) Vuill. (Cordycipitaceae: Hypocreales). Plant protection from arthropod pests by using EEPF can occur by deterring feeding, slowing insect growth, reducing both survival and oviposition or a combination of these mechanisms [2,4,7,8,9,10,11]. It is because of the effectiveness of these properties and mechanisms for protecting crop plants that EEPF have become of interest to agricultural researchers to utilize as part of an integrated pest management strategy. However, because information about the host–plant relationships is lacking, there are few reliable methodologies for utilizing EEPF that have been evaluated in an agricultural setting [6,8,12,13].

Endophyte inoculation methods, species of fungi tested, and host plants were catalogued by Vega [2]. The most often used methods include soil/root treatment, foliar spray, and seed coating/soaking. However, few studies have compared the different methodologies Sánchez-Rodríguez et al. [14] found that *B. bassiana* colonization was more successful through seed coating and soil treatment compared to foliar spray in wheat (*Triticum* spp.). Russo et al. [15] found foliar spray to be more effective than seed inoculation or root immersion, but all resulted in transient colonization in tobacco (*Nicotiana tabacum* L.), corn (*Zea mays* L.), wheat, and soybean (*Glycine max* L.). Posada et al. [12] compared soil drench, foliar spray, and stem injection with *B. bassiana* on coffee (*Coffea arabica* L.) and found that plants were successfully inoculated through all three methods, but stem injection was the most effective. Recent research suggests that successful colonization of a host plant by EEPF is dependent on inoculation methods in combination with edaphic ecological factors [16].

Vega [3] also demonstrated the limited breadth of fungal species studied for endophytism. Research evaluating agriculturally beneficial endophytes has been conducted with a wide variety of crops but few entomopathogenic fungi. *Beauveria bassiana*, *Akanthomyces* (formerly *Lecanicillium*) *lecanii* (Zimm.) Spatafora, Kepler and B. Shrestha (Cordycipitaceae: Hypocreales), and *Metarhizium* species have been the primary endophytes selected for these types of studies [8,12,17]. In addition, these afore mentioned EEPF are already commonly applied as insect pathogenic biopesticides, and their establishment within the agricultural industry makes them logical choices for studying endophytism. However, *Cordyceps fumosorosea* (Wize) Kepler, B. Shrestha and Spatafora (Hypocreales: Cordycipitaceae) (*Cfr,* formerly *Isaria fumosorosea*) is also commonly applied as a fungal biopesticide, and one of a few species that have been found to naturally infect plants [3,18,19]. For instance, *Cfr* colonizes eggplant (*Solanum melongena* L.) [20] and sorghum, *Sorghum bicolor* L. (Moench) [21].

In the southern United States, citrus production is well-established, but the citrus industry is now struggling to survive due to huanglongbing (HLB; citrus greening). Techniques to protect citrus trees from arthropod pests like the Asian citrus psyllid, *Diaphorina citri* Kuwayama (Hemiptera: Liviidae), which is the insect vector associated with HLB [22,23], and the citrus root weevil, *Diaprepes abbreviatus* (L.) (Coleoptera: Curculionidae), which is the insect vector associated with the transmission of the oomycete plant pathogen *Phytophthora* spp. [24,25,26], are in high demand. Thus, researchers have begun investigating the use of endophytes as a potential management strategy for plant protection. When testing foliar sprays of *B. bassiana* and *Cfr* on *Citrus limon* (L.) Osbeck, Bamisile et al. [27] found that two strains of *B. bassiana* were able to successfully colonize the plant, but *Cfr* was unsuccessful. There are no other publications about endophytic *Cfr* in citrus plants.

To determine if *Cfr* is capable of colonizing *Citrus* species, a commercially available cultivar [Carrizo citrange (*Citrus × insitorum* Mabb.)] used as a rootstock was chosen. In this study, four different inoculation methodologies were tested: seed soaking, stem injection, foliar spray, and soil drench. It was hypothesized that *Cfr* would be capable of becoming endophytic and persist over an extended period of time.

## 2. Materials and Methods

### 2.1. Seed Sterilization

Carrizo citrange (*Citrus × insitorum* Mabb.) seeds (Lyn Citrus Seed Inc., Arvin, CA, USA) were stored at 4 °C to prevent germination until needed. After removal from the refrigerator, seeds were surface sterilized by immersion in 0.5% sodium hypochlorite solution for 2 min, followed by a 2 min immersion in a 70% ethanol solution, and then rinsed with sterile autoclaved distilled water 3 times. To ensure the sterilization technique was successful, aliquots of 100 μL of the final rinsate were spread on 5 sterile plastic Fisherbrand^®^ (Fisher Scientific, Hampton, NH, USA) Petri dish plates (100 × 15 mm) containing potato dextrose agar (PDA: Difco^TM^, Becton, Dickinson & Co., Sparks, MD, USA), with streptomycin sulfate and chloramphenicol [12,28]. PDA plates were sealed with Parafilm™ M (Bemis Co., Neenah, WI, USA) and then placed in a growth chamber at 25 °C under a 14 h light (L): 10 h dark (D) photoperiod. The PDA plates with the final sterilization rinsate were examined for contamination two weeks after plating.

### 2.2. Fungal Inoculum Preparation

One gram of PFR-97 20% WDG (Certis USA, Columbia, MD, USA) containing blastopores of *Cfr* was added to 100 mL of distilled water and mixed with a magnetic stirrer for 30 min. Afterwards, the suspension was given 30 min to settle. From the supernatant, 10 μL was spread onto PDA plates with a flame sterilized bent glass rod. Plates were sealed with Parafilm™ and placed in the growth chamber as described above. This process was repeated 10 times. After allowing 2–3 weeks for conidiation of *Cfr*, the plates were flooded with 0.1% Triton X-100 (solution mixed with phosphate-buffered saline [12,29,30]. The fungal suspensions from two cultured plates were poured through 4 pieces of sterile cheesecloth covering the top of a 100 mL beaker. After pouring two suspensions into one beaker, the conidial concentration was determined using a C-Chip disposable hemocytometer (INCYTO Co., Ltd., Cheonan-si, Chungnam-do, Republic of Korea) to be at least 2 × 10^8^ conidia mL^−1^. Conidial concentrations were adjusted to 10^7^ conidia mL^−1^ for the seed inoculation experiment and 10^8^ conidia mL^−1^ for the plant inoculation experiment [12,27].

### 2.3. Seed Inoculation

Fourteen days after seed germination was initiated, 46 citrange seeds were randomly assigned to the treatment or control group. The 23 seeds assigned to the treatment group were submerged in a 60 mL conidial suspension of 2 × 10^7^ conidia mL^−1^, while the other 23 seeds assigned to the control group were submerged in 60 mL of 0.1% Triton X-100. Each beaker was then sealed with Parafilm™ and placed in the growth chamber as described above. After 24 h, the seeds were transferred into plastic germination boxes (SunShine Industries, Haryana, India; 13 × 13.5 × 3.5 cm) and kept moist with sterilized brown paper towels placed on the bottom.

After two weeks, seeds from each treatment were removed from their respective germination boxes. Their roots were cut and sterilized the same process as the seed sterilization process described above [12]. From the last rinsate, 100 μL was spread on 5 PDA plates, sealed with Parafilm™ and the plates were transferred to the same growth chamber at 25 °C under a 14:10 h L:D photoperiod. After 14 days, plates were checked for contamination to ensure sterilization was successful. The sterilized root ends were removed, and the rest of the root was cut into 2 or 3 approximately 7 mm segments and placed on PDA plates [12]. Plates were then sealed with Parafilm™ and transferred to the growth chamber for 14 d as described above. The criteria used for successful infection, colonization and morphological confirmation of the fungi, were that the phenotype of the *Cfr* fungus was observed growing on the PDA plate only out of the cut root section ends compared to the control plates.

This entire experiment was then repeated, using another batch of 46 seeds, resulting in a total of 92 seeds. However, some seeds were excluded from further analyses due to unsuccessful sterilization of the roots during plating that prevented seeds from being processed (*n* = 74).

### 2.4. Plant Inoculation

Prior to inoculations on leaves or stems, young plants were obtained from seeds. Seeds were potted in 7.6 L pots (Black Thermoformed Nursery Pot) containing pure play sand (Sakrete of North America LLC., Charlotte, NC, USA). Pots were fertilized with Hoagland complete medium (Bio-World, Dublin, OH, USA) once every two weeks and watered as needed. After one month, 48 seedlings were selected and transplanted into 10.2 × 10.2 cm pots containing sand and allowed a week to reestablish. Plants were then randomly assigned to one of three treatment groups or one of three control groups: foliar spray control, foliar spray with *Cfr*, stem injection control, stem injection with *Cfr*, soil drench control, and soil drench with *Cfr*. Control treatments received 0.1% Triton X-100 solution, while *Cfr* treatments received 0.1% Triton X-100 + PFR-97 20% WDG suspension.

For stem injection inoculations, a sterile 21 G hypodermic needle on a Hamilton syringe (Hamilton, Reno, NV, USA) was used to inject 0.4 mL of 0.1% Triton X-100 + PFR-97 20% WDG suspension or 0.1% Triton X-100 solution (control). Plants in the soil drench treatment received 4 mL of 0.1% Triton X-100 + PFR-97 20% WDG suspension or 0.1% Triton X-100 solution (control) applied to the surface of the soil in each treatment pot. Plants receiving the foliar spray treatment had aluminum foil placed around the base of the pot to avoid soil contamination. A Nalgene^®^ hand pump sprayer (Nalgene Nunc International, Rochester, NY, USA) was then used to apply 2 mL of 0.1% Triton X-100 + PFR-97 20% WDG suspension or 0.1% Triton X-100 solution (control) per plant. After inoculation, all plants were covered with a plastic bag for 24 h to maintain a high level of humidity and facilitate fungal establishment. There were 2 trials conducted, each with 48 plants, resulting in a total of 96 plants for this experiment.

### 2.5. Plant Physical Attributes and Assessment

**Figure 1:**
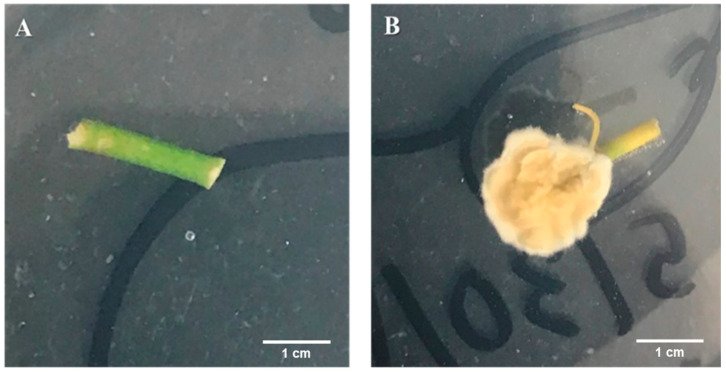
Segments of citrus stems showing endophytic growth of *Cordyceps fumosorosea* on the potato dextrose agar (PDA) plates. (**A**) Control stem piece with no colonization after inoculation with water treatment. (**B**) Inoculated stem piece with the endophytic *C. fumosorosea* fungus with mycelium at the stem piece end after 14 days post-inoculation.

Plant physical attributes (stem diameter, number of leaves, and plant height) were measured one month after inoculation. Afterwards, plants were uprooted and individually washed with running tap water. The root, stem, two top leaves, and two middle leaves were surface sterilized as described above [12]. To ensure each sterilization was successful, 100 μL of the final rinsate was spread on PDA plates, sealed with Parafilm™, placed in the growth chamber as described above and examined for fungal contamination 14 days later. The plant parts were cut with sterile scalpels and forceps. Leaves were cut into 2 × 3 mm rectangles, while roots and stems were cut into 7 mm length pieces starting from the root tip and moving upward towards the intersection of the root-stem. Pieces were placed on PDA plates that were then sealed with Parafilm™ and incubated, as indicated above. Pieces were observed 14 days later to determine if endophytic colonization of *Cfr* had occurred in planta (Figure 1).

### 2.6. Sand Core Assessment

To determine conidial concentrations of PFR-97 at different depths of the soil drench treatment, the methods of Avery et al. [31] were followed. Plastic tips of 25 mL pipettes (Fisher Scientific, Inc., Hampton, NH, USA) were modified by cutting off at the 2 mL mark on the pipette’s tip. Core samples were taken from each pot with PFR-97 by pushing the modified pipettes into moist sand to the bottom of the pot. The modified pipette was then carefully removed to ensure that the sand remained inside it. Pipettes containing sand cores were then cut with a buzz saw in pieces of 1.5 cm increment, 3 times at each consecutive 2 mL mark. Each of the 1.5 cm core samples per depth (1.5, 3.0, 4.5 cm) was poured into plastic bags and each bag contained 8 sand core samples from the same depth. After the sand cores in each bag were shaken, 10 g of sand was collected from each bag and placed in a 50 mL centrifuge tube (Fisher Scientific, Inc., Hampton, NH, USA) containing 30 mL of distilled water. Each tube was shaken by hand and vortexed for 30 s. After the sand precipitated to the bottom of the tube, conidial concentrations in the supernatant were assessed with a C-Chip hemocytometer and adjusted to 10^4^ conidia mL^−1^. From the supernatant suspension, aliquots of 100 μL were spread using a flame sterilized glass rod on sterile plastic Fisherbrand^®^ Petri dish plates (100 × 15 mm) containing PDA-dodine (modified as a selective medium for entomopathogenic fungi), streptomycin sulfate and chloramphenicol. Plates were sealed with Parafilm™ and transferred to a growth chamber under the same conditions as described above. The number of colony-forming units (CFUs) per depth was counted after 2 weeks incubation time.

### 2.7. Statistical Analysis

Experimental design was randomized complete blocks, blocked by trial replicates. Chi-square analyses (α = 0.05) were used to determine inoculation success rates, and for trial replicate effects (α = 0.05). The plant attribute analysis only had one experimental replicate, making the design a completely randomized design interpreted through a multivariate analysis of variance (MANOVA; α = 0.05). Student’s *t*-tests (α = 0.05) were used for pairwise comparisons for assessing plant attributes between treatments. The sand core experiment was also a randomized complete block design [31]. A one-way analysis of variance (ANOVA; α = 0.05) was conducted for the sand core assessment by using trial replicate as a random effect. Student’s *t*-tests (α = 0.05) were used for *post-hoc* pairwise comparisons between the number of CFUs per sand core depth. Statistical tests were conducted using JMP 15 (SAS Institute, Cary, NJ, USA).

## 3. Results

### 3.1. Seed Inoculation

There was no effect amongst replicate trials (χ^2^ = 2.37; df = 1, *n* = 74; *p* = 0.12); therefore, the treatment groups were combined for analysis. Seeds that were soaked in *Cfr* for 24 h, 28% of them were successfully inoculated (χ^2^ = 15.81; df = 1, *n* = 74; *p* < 0.0001) compared to the control. No seeds in the control group contained *Cfr*. Successfully inoculated seeds displayed *Cfr* in different sections of the root. The frequency of endophytic fungus displayed was 72% in the top (apical) section of the root, and 27% in the tip (basal section) of the root. Not every sample had a long enough root to be cut into apical, middle, and basal sections. However, of the inoculated roots that were long enough to be cut into three pieces, 57% displayed *Cfr* in the middle section.

### 3.2. Plant Inoculation

**Figure 2:**
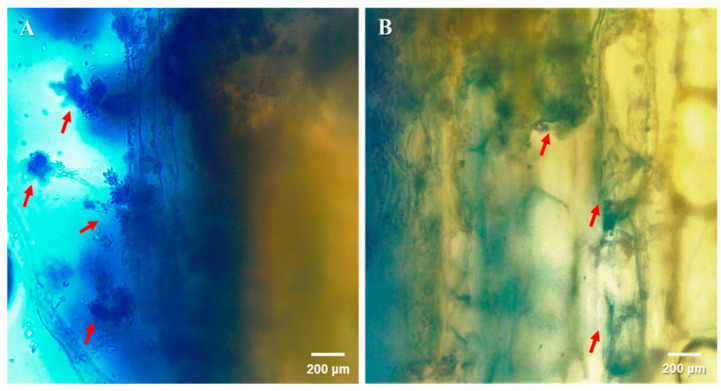
Localization of *Cordyceps fumosorosea* (*Cfr*) outside (**A**) and inside (**B**) citrus stem tissues. Samples were excised from the top of a 3 plant stems in the stem injection treatment using a sterile free-hand razor blade technique. Specimens were then placed on a single microscope slide and a drop of trypan blue (0.4% *w*/*v*) was used as a contrast dye. Images were then obtained using an optical video light microscope (Leica DM750: Wetzlar, Germany) at the magnification of 40×. Arrows indicate *Cfr* propagules observed outside and inside the tissue. These observations represent only the injection treatment.

Overall, of the plants inoculated with *Cfr*, 22% were colonized by the fungus (χ^2^ = 18.77; df = 1, *n* = 48; *p* = 0.002); none of the control plants contained *Cfr*. Among the fungus-treated plants, 31.3%, 26.7%, and 12.5% contained *Cfr* in the stem injection, foliar spray, and soil drench treatments, respectively. However, there were no significant differences among these three methods (χ^2^ = 2.14; df = 3, *n* = 96; *p* = 0.34). There was also no effect amongst trials (χ^2^ = 0.93; df = 2, *n* = 96; *p* = 0.33). Regardless of the plant inoculation method, *Cfr*, when present, was always found in the stem of the plant (Figure 2), never in the roots or leaves of the plant.

Plants showed no significant differences in their number of leaves, plant height, or stem diameter based on the presence of *Cfr* or application methodologies (i.e., foliar spray, stem injection, soil drench). Moreover, there was no interaction effect between methodology and treatment, indicating that plant traits were not affected by *Cfr* presence within any single methodology (Table 1).

### 3.3. Plant Physical Attributes and Assessment

There were no significant differences in the number of leaves (*t* = −0.20, *p* = 0.84), plant height (*t* = −0.52, *p* = 0.61), or stem diameter (*t* = 0.95, *p* = 0.34). These results were regardless if the plant was successfully colonized or not.

### 3.4. Sand Core Assessment

**Figure 3:**
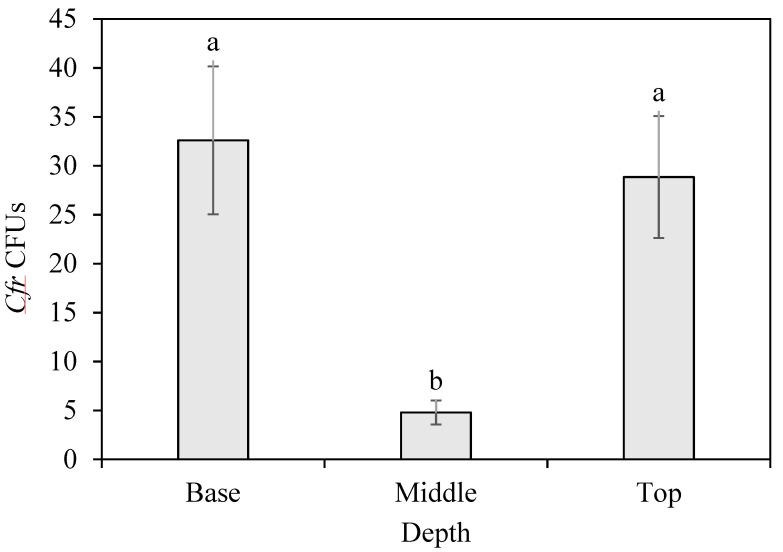
Number of *Cordyceps fumosorosea* colony-forming units (*Cfr* CFUs) counted on PDA-dodine plates after inoculation with extracts from three depths of the sand profile. Depth per sand core stratum in inoculated pot: Base = 4.5 cm; Middle = 3.0 cm; Top = 1.5 cm. Letters above the ±SE bars per depth that are not the same are significantly different (Student’s *t*-tests, *p* < 0.05).

There were significant differences (*F*_2, 56_ = 26.75; *n* = 60; *p* < 0.0001) in the number of CFUs across core strata in the soil drench treatments (Figure 3). The bottom layer (CFUs = 32.6 ± 7.55) and the top layer (CFUs = 28.9 ± 6.23) had significantly higher CFU counts compared to the middle layer (CFUs = 4.8 ± 1.23), respectively (*t* = 5.83, *p* < 0.0001; *t* = 6.74, *p* < 0.0001). The top and bottom layers were not significantly different from each other (*t* = 0.91, *p* = 0.18).

## 4. Discussion

Since Carrizo citrange is used as rootstock, the ability to inoculate its roots could provide potential pest management benefits, particularly against the citrus root weevil. Also, because *Cfr* is an effective fungal biopesticide used for management of various citrus pests, including *D. abbreviatus* [32], this EEPF was tested to determine if it demonstrated endophytism in citrus roots as well as other parts of the plant. Seed immersion trials demonstrated that the roots of the plant can be inoculated successfully with *Cfr*. This is the first report of *Cfr* showing endophytic properties in Carrizo citrus plants. Further research might investigate the longevity of *Cfr* endophytism following seed immersion, as well as its efficacy as a pest management tool against *D. abbreviatus*.

For whole plant tests, *Cfr* was successfully inoculated in Carrizo citrange. Each method (stem injection, foliar spray, and soil drench) provided successful inoculation of *Cfr* as an endophyte *in planta*. However, *Cfr* was endophytic only in the plant stem, and stem injections had the greatest success rate among the inoculation methods, although the rate was not significantly different from those by the other two methods. Borisade [33] demonstrated endophytic colonization of *Cfr* in sorghum roots, stems. and leaves. Graham et al. [34] reported that plant nutrients are primarily accumulated in the stem. Therefore, it could be that the stem is where *Cfr* is most capable of utilizing the nutrients it needs to develop.

Alternatively, Carrizo citrange may be treating *Cfr* as a pathogen, and it compartmentalized the fungus into the stem. This is a common defense mechanism in plants [35] and has been observed for various pathogens [36,37]. If this was the case in our study, we would no longer consider *Cfr* endophytic because of its inability to move throughout the plant. Our study was limited to the span of a month, and in that time frame, *Cfr* appeared to be endophytic. Longer term studies are needed to determine if it is truly endophytic throughout the plant’s lifetime. Interestingly, Borisade [33] was able to demonstrate that *Cfr* could be endophytic *in planta* with sorghum plants within a similar time frame compared to our study.

Our experiments broadly followed the procedures described by Posada et al. [12], except they used *B. bassiana* as an inoculum to determine endophytism in coffee plants. In our study, *Cfr* was used as an inoculum to determine endophytism in citrus plants. While the inoculation methods employed were similar, the results were different. For instance, Posada et al. [12] found that inoculation method affected where EEPF localization occurred. Conversely, we found that *Cfr* resided only in the stem of whole plants, regardless of the inoculation method. *Cfr* only appeared outside of the stem after seed immersion, while Bamisile et al. [27] found *Cfr* to not be endophytic in *C. limon*. Each of these studies is unique among the research, which may suggest these differences arise due to the host plant.

Various field studies have shown that *Cfr* is effective against the Asian citrus psyllid when applied as an innundative spray [38,39]. However, very little research has focused on this fungus being used as an EEPF [27]. In contrast to our findings, Bamisile et al. [27] observed that the *Cfr* strain IF Fafu-1 was unsuccessful in colonizing the *C. limon* seedling after spraying the foliage. Since the only inoculation method employed by Bamisile et al. [27] to encourage colonization of *Cfr* was via an innundative spray, the other inoculation methods we tested (i.e., seed immersion, stem injection, soil drench) cannot be compared, thus warranting further investigation with other *Citrus* species.

Through our sand core assessments, we determined that *Cfr* moved down through the sand column and was able to inoculate the roots; however, *Cfr* did not remain inside the roots as an EEPF. This finding corroborated with that of Avery et al. [31], where *Cfr* was able to move through the sand as the water drench amount increased. In contrast to Avery et al. [31], our study differed in that the *Cfr* fungal inoculum contained conidia, not blastospores and the water drench continued on a daily basis for a month, as compared to only a single drench for < 1 min. Considering *D. abbreviatus* feeds on the roots of citrus plants, but *Cfr* was not localized in the roots of Carrizo citrange, it is unlikely for this fungus to act as a reliable biocontrol against this pest as an EEPF through whole plant inoculation of this citrus plant. Moreover, while *Cfr* has been demonstrated as an effective biological control of *D. abbreviatus* adults, it is less effective against the soil dwelling larvae [31]. It is unclear exactly why larval mortality by *Cfr* is relatively low. Nevertheless, given the prevalence of *Cfr* in the soil during the drench experiment and our finding that *Cfr* did colonize Carrizo citrange roots through seed immersion, there are still several questions worth investigating.

## 5. Conclusions

Given the prevalence of *Cfr* in the soil during the drench experiment, and that the fungus was able to colonize Carrizo citrange roots through seed immersion, this finding provides evidence of the potential endophytism of this fungus when applied to citrus plant species. Future researchers might examine if the endophytic association of *Cfr* in Carrizo citrange roots seen in the seed immersion experiment remains once seeds are planted in soil and the plants develop. This can be particularly important for breeding programs looking at ameliorating root traits in citrus rootstocks [40]. The use of *Beauveria*-based products that may be more endophytic in the roots can be used against both the Asian citrus psyllid [41] and *Diaprepes* root weevil [25]. Additionally, while *Cfr* may be less effective against *D. abbreviatus* larvae than against adults, the mechanisms behind that difference should be closely examined. If it can be circumvented, soil drenches of *Cfr* may still be an effective control method, given its prevalence within the soil profile during our experiments. Finally, revealing why *Cfr* localizes in the stems of whole plants may assist in refining inoculation methods and improving *Cfr*’s EEPF potential.

## Figures and Tables

**Table 1:** Multivariate analysis of variance (MANOVA) of physical traits and potential interactions with inoculation methods (leaf spray, soil drench and stem injection), inoculation treatment (fungus or control), and inoculation success.

Traits	Source	*df*	*F* Ratio	*p*-Value
Plant Height	Inoculation Method	2	0.8125	0.4508
	Inoculation Treatment	1	0.3650	0.5491
	Inoculation Success	1	0.1183	0.7327
	Method × Treatment	2	0.5684	0.5708
Leaf Number	Inoculation Method	2	1.5907	0.2161
	Inoculation Treatment	1	0.0071	0.9335
	Inoculation Success	1	0.0338	0.8549
	Method × Treatment	2	1.2393	0.3002
Stem Diameter	Inoculation Method	2	0.1704	0.8440
	Inoculation Treatment	1	0.7258	0.3992
	Inoculation Success	1	0.3098	0.5808
	Method × Treatment	2	0.3484	0.7079

## Data Availability

The data presented in this study are available on request from the corresponding author.
